# Blood pressure control status of patients with hypertension on treatment in Dessie City Northeast Ethiopia

**DOI:** 10.1186/s12889-022-13368-6

**Published:** 2022-05-09

**Authors:** Zinabu Fentaw, Kidist Adamu, Shambel Wedajo

**Affiliations:** 1 Department of Epidemiology and Biostatistics, School of Public Health, College of Medicine and Health Sciences, Wollo University, Dessie, Ethiopia; 2 Department of Health Service Management, School of Public Health, College of Medicine and Health Sciences, Wollo University, Dessie, Ethiopia

**Keywords:** Blood pressure, Hypertension, Prevalence ratio, Uncontrolled, Ethiopia

## Abstract

**Introduction:**

Uncontrolled blood pressure contributes a huge contribution to many hypertension-related complications and it is one of the unbeaten problems for patients taking antihypertensive drugs. The association of social support and other factors with uncontrolled blood pressure during the covid-19 pandemic is not well investigated. Therefore, this study explored the determinants of blood pressure control status during the COVID-19 pandemic among patients with hypertension who were on an antihypertensive treatment.

**Method:**

A cross-sectional study was done from March to May 2021 among adults aged 18 or more patients with hypertension for three months or more on treatment in Dessie City. An interview-administered questionnaire was done using simple random sampling from hypertension follow-up register for 380 patients with hypertension. Blood pressure measurement was taken from their arm using a stethoscope and mercury sphygmomanometer at a sitting position with 90-degree back support. Uncontrolled blood pressure was also computed either the systolic or diastolic blood pressure greater than or less than the limit of uncontrolled blood pressure with regarding the age and diabetic status of patients. The perceived social support-related questionnaire was adopted from the Multidimensional Scale of Perceived Social Support (MSPSS) -12 item checklist. It was sum-up and transformed into three categories using tertile of their computed raw scores. The adjusted prevalence ratio with a 95 percent confidence interval (CI) was used to calculate the strength of the association between uncontrolled blood pressure and independent predictors using log-binomial regression analysis. A *P*-value less than 0.05 was declared as statistically significant in multivariable log-binomial regression analysis.

**Result:**

A total of 360 study participants were included in this study. The prevalence of uncontrolled blood measures in patients with hypertension with a 95% CI was 55.8(50.7, 61.0). In a multivariable analysis adjusted prevalence ratio with 95% CI for poor medication adherence 1.86(1.59,2.19), being male 1.35(1.11,1.64), secondary education 0.52(0.35,0.77), and low social support 1.24(1.01, 1.54) were the predictors of uncontrolled blood pressure.

**Conclusion:**

Uncontrolled blood pressure for patients with hypertension on treatment is higher during the COVID-19 pandemic. Being male, poor medication adherence, educational status and low social support are factors that contribute to uncontrolled blood pressure.

## Introduction

High blood pressure (BP) remains the leading cause of premature death globally, accounting for 10.4 million deaths per year [1]. The global estimate showed that 1.39 billion people had hypertension in 2010 [2]. However, blood pressure trends reveal a significant movement of the highest blood pressure from high-income to low-income regions [3]. Between 2000 and 2010, the prevalence of hypertension in high-income nations fell by 2.6 percent, and awareness, treatment, and control increased significantly. Low- and middle-income nations reported a 7.7 percent rise in prevalence over the same 10-year period, with little progress in knowledge, treatment, and control [2].

Blood pressure control is the main goal of hypertension treatment and follow-up [4]. Even after the initiation of treatment patients with hypertension face life-threatening complications and death due to the significant contribution of uncontrolled blood pressure [5]. Most of the common complications faced in raised blood pressure patients are heart attack, stroke, congestive heart failure, and kidney disease. Additionally, inadequate blood pressure control increases the risk of hypertensive crises with the need for hospitalization [6].

Uncontrolled blood pressure in hypertension is one of the modifiable diseases [5] and most of the factors affecting uncontrolled hypertension are known. Medication adherence, age, sex, level of education, marital status, behavioral factors, and obesity are some of the contributing factors to blood pressure control status, but these are not the only factors for poor control of blood pressure status [7–11]. Poor blood pressure control status is one of the unbeaten problems in the current implementing system of hypertension treatment [12]. The social support status, smoking, and khat chewing practice are not well investigated in the context of blood pressure control status of patients with hypertension on treatment. Hypertension patients need frequent follow-ups for measuring their blood pressure and adjusting their medication [13], which is difficult in pandemics of COVID-19. During this epidemic, not just chronic patients, but all residents of the country, are restricted from moving and socializing. Moreover, patients with hypertension fail to control their blood pressure level at the set of standards in the era of COVID-19. So, this study investigates the magnitude of uncontrolled BP and factors that contribute to uncontrolled blood pressure during this pandemic.

## Methods

### Study design and settings

A cross-sectional study was done from March to May 2021 among adults aged 18 or more patients with hypertension for three months or more on treatment [14] in Dessie City. Patients were treated in eight health facilities with different health care system levels. One Comprehensive Specialized Hospital, one Generalized Specialty Hospital, and six health centers within the district of the city. There were more than 2109 patients with hypertension on treatment in the city at the end of January 2021. The national guideline recommends for patients with hypertension visit the health facility on monthly basis by checking their blood pressure status at each visit and modification of drugs according to the recommended guidelines [13]. But the guideline was violated in the era of COVID-19 and amended as patients visit the health facility every three months or more. Adult patients with hypertension active on treatment were considered for inclusion in this study. Since chronic hypertension is a risk of high blood pressure during pregnancy [14], pregnant women were excluded from this study.

### Sample size determination and sampling procedures

The largest sample size was obtained using a single population proportion formula from the study conducted in Southwest Ethiopia with 49.7% of uncontrolled blood pressure including an assumption of 95% level of Confidence and 5% of errors [9]. So, the final sample size of this study was 380 patients with hypertension. Participants were recruited into the study using simple random sampling methods. Study participants were listed from the hypertension register book with serial numbers for each facility then a computer-generated lottery method was employed from serial numbers.

### Data collection tools and measurements

Most of the data were collected using structured interviewer-administered questionnaires, which were prepared to address all the important variables with supportive document review using patients’ charts after obtaining informed consent to immediately exit from their clinical care. The questionnaires were adapted from different kinds of literature of similar studies [8–10, 15]. The perceived social support-related questionnaire is adopted from the Multidimensional Scale of Perceived Social Support (MSPSS) -12 item check [16] and it was validated with a similar setting [17]. Each of these items was scored from 1 to 5 on a response scale, which agreed as a five-point Likert scale. Assessment of Anthropometric Measurements for Height (cm) and weight (kg) were measured for all participants using standard methods [18] Additionally, body mass index (BMI) was estimated by dividing weight (kg) by the square of height in meters. The BP measurement was taken from the left arm (mmHg) using a stethoscope and mercury sphygmomanometer at a sitting position with 90-degree back support. The arm is bare and the middle of the cuff at the heart level with uncrossed legs without talking during and between measurements. An average level of the two consecutive measurements (Average 2^nd^-3^rd^ measurement) was taken as the accurate measurement [14]. In this study, Uncontrolled BP was defined according to the Eighth Joint National Committee (JNC-8) definition, as a systolic BP equal to or above 140 mmHg and/or diastolic BP equal to or above 90 mmHg after taking treatment of three months or more for patients with diabetes chronic kidney diseases and adults aged less than 60 years old. Whereas, for patients older than 60 years uncontrolled blood pressure is defined as above 150/90 mmHg of their systolic/diastolic blood pressure [19]. Adherence means accepting, agreeing, and correctly following a prescribed treatment [13]. The adherence status of the respondents was assessed using patients' self-report. So, poor adherence is missing 20% or more of the monthly prescribed doses based on patient reports.

### Data processing and analysis

Data were checked for completeness, then entered into Epi Data version 3.1 and exported to STATA/SE 14.0 for further analysis. Also, the data were coded, cleaned, and explored to identify missing values, outliers, and inconsistencies through tabulation and graphical display.

The blood pressure measurement was taken as a continuous variable, but for the sake of understanding it compute as controlled when both systolic and diastolic blood pressure amounts were less than the limit of uncontrolled blood pressure by considering age and diabetes mellitus. Uncontrolled blood pressure was also computed either the systolic or diastolic blood pressure was greater than the limit of uncontrolled blood pressure with regarding the age and diabetic status of patients.

The perceived social support was also transformed into three categories using tertile of their computed raw scores. The lowest, middle and highest tertiles were recoded as low, moderate, and high social supports, respectively [16]. Continuous variables were described in terms of mean with their standard deviation and median with their interquartile range. Categorical data were also described in terms of frequencies and percentages. All necessary assumptions of log binomial regression model were checked and fulfilled. Those variables in the bivariable log binomial model regression analysis with a *p*-value less than 0.25 were transferred to multivariable log binomial model [20]. Then, a multivariable log binomial regression analysis was done and those variables whose *p*-value was less than 0.05 at a 95% CI were declared statistically significant.

### Ethical issues

Ethical clearance was obtained from the Ethical Review Committee of Wollo University College of Medicine and Health Sciences. The respondents were informed about the purpose of the study, and written consent was obtained from each participant. The data were collected in accordance with the Helsinki declaration. The respondents’ right to refuse or withdraw from participation in the interview is fully maintained and the information provided by each respondent was kept strictly confidential.

## Result

### Sociodemographic, clinical, and behavioral risk of the study participants

A total of 360 study participants were included in this study. The median age of the respondents was 55 years with an interquartile range of 17 years old. The minimum and maximum ages of the participants were 20 years old and 88 years old, respectively. One hundred fifty-three (28.3%) of the participants were males. Regarding marital status and residence of the participants 243(67.5%) and 258(71.67%) were married and urban dwellers, respectively. Concerning participants' education and occupational status, 226(62.78%) and 160(44.4%) have no formal education and were government-employed, respectively (Table 1).

**Table 1:** Socio-demographic, clinical, and behavioral characteristics of patients with hypertension in Dessie City, 2021

Variables	Category	Uncontrolled n (%)	Controlled n (%)
Sex	Male	102(28.3)	51(14.2)
Marital status	Currently Married	139(38.6)	104(28.9)
	Currently Not Married	62(17.2)	55(15.3)
Educational Status	Have no Formal Education	121(33.6)	105(29.2)
	Primary	15(4.2)	23(6.2)
	Highschool and above	65(18.1)	31(8.6)
Residence	Urban	146(40.6)	112(31.1)
Occupational Status	Government	78(21.7)	82(22.8)
	Private	43(11.9)	23(6.4)
	Self-employed	55(15.3)	42(11.7)
	Unemployed^a^	25(6.9)	12(3.3)
Complication	Yes	70(19.4)	60(16.7)
Symptoms	More than one	176(48.9)	127(35.3)
Number of drugs	Two or more drugs	134(37.2)	101(28.1)
Medication Adherence	Poor	26(7.2)	2(0.6)
Comorbidity^b^	Yes	78(21.7)	53(14.7)
Smoking	Yes	8(2.2)	6(1.7)
Khat Chewing	Yes	19(5.3)	15(4.2)
Drinking Alcohols	Yes	13(3.6)	8(2.2)
Physical Exercise	No	53(14.7)	55(15.3)
Salt intake	Not restricted	31(8.6)	20(5.6)
Social Support^c^	Low	48(13.3)	37(10.2)
	Moderate	90(25.0)	53(14.7)
	High	63(17.5)	69(19.2)

^a^Unemployed: daily laborer, Housewife, and students

^b^Comorbid illness is defined as any condition that is not the direct cause or result of hypertension, such as HIV or cancer

^c^Social Support = Low social support: participants whose total social support score is below 33% of the total score. Moderate Social support: participants whose total social support score is between 33 and 67% of the total score. High social support: participants whose total social support score is above 67% of the total score

The prevalence of uncontrolled blood measures in patients with hypertension with 95% CI was 55.8(50.7, 61.0). The clinical characteristics of the study participant showed 130(36.1%) patients with hypertension had at least one complication of hypertension. Most of the patients 303(84.2%) had one or more hypertension-related symptoms and 235(65.3%) of the participant also took two or more hypertensive drugs. The presence of comorbidity and adherence status 131(36.4%) and 28(7.8%) of patients with hypertension had one or more co-morbidity and poor adherence, respectively. The mean body mass index (BMI) of the participant was 23.6 kg/m^2^ with a standard deviation of 3.4 kg/m^2^. The median stay of the patient with antihypertensive treatment was 6 years with an interquartile range of 7 years (Table 1).

Only fourteen (3.9%) of patients with hypertension were smoking cigarettes. Considering alcoholic consumption and ‘Khat’ chewing practice 21(5.8%) and 34(9.4%) of patients with hypertension were also drinking alcohol and Chewing ‘Khat’, respectively. For the habits of physical exercise and salt restriction, 252(70%) and 51(14.2%) of the patients were performed regular physical exercise and unable to restrict their salt intake, respectively. The perceived social support status of participants 85(23.6) had a low perceived social support (Table 1).

### Factors affecting uncontrolled blood pressure

Due to a high prevalence of uncontrolled blood pressure, we chose PR over POR because POR would have significantly "overestimated" the strength of the association [21]. Some modifiable and non-modifiable factors were assessed for the presence of associations with blood pressure control status in multivariable log binomial model and the following variables were significantly associated.

Male patients with hypertension were 1.35 (1.11,1.64), times more likely to be uncontrolled blood pressure than those female patients with hypertension. The odds of having uncontrolled blood pressure in poor medication adherence patients were 1.86(1.59,2.19) times higher than in patients with good medication adherence. Those patients with hypertension with educational status of secondary and above were 48% [0.52(0.35,0.77)] less likely of having uncontrolled blood pressure than patients with no formal education. Patients with hypertension on treatment, who had low support were 1.24(1.01, 1.54) times more likely of having uncontrolled blood pressure than those patients with hypertension with high social support (Table 2).

**Table 2:** Factors affecting uncontrolled blood pressure of patients with hypertension in Dessie City, 2021

Variables	APR (95% CI)	*P*-value
**Sex**		
Male	1.35(1.11,1.64)	0.002*
**Age**	1.00(0.99,1.01)	0.203
**Education Status**		
No Formal Education	1	1
Primary	1.09(0.94,1.16)	0.071
Secondary and above	0.52(0.35,0.77)	0.001*
**Medication Adherence**		
Poor	1.86(1.59, 2.19)	< 0.001*
**Comorbidity**		
Yes	1.01(0.98, 1.02)	0.17
**Duration of treatment**	1.00(0.99, 1.02)	0.072
**Number of drugs**		
Two or more drugs	1.05(0.86, 1.29)	0.587
**Khat Chewing**		
Yes	1.11(0.79, 1.56)	0.527
**Physical Exercise**		
Yes	0.85(0.66, 1.08)	0.183
**Social Support**		
Low	1.24(1.01, 1.54)	0.048*
Moderate	1.22(0.88, 1.41)	0.33
High	1	1

^*^Statistically significant variables

## Discussion

The primary target of this study is to investigate the prevalence of uncontrolled blood pressure status and factors that contribute to it for patients on treatment for three months or more during the pandemics of COVID-19.

In this study, more than half (55.8%), of the participants had uncontrolled blood pressure. This is in line with a cross-sectional study conducted in Southwest, Ethiopia, Korea, and Iran, [9, 22, 23] while this result is significantly lower than the study conducted in Nigeria and United States [8, 24] This finding is also higher than a study conducted in Nigeria [10] and Gondar, Ethiopia [15]. The reason for being higher than these studies may be, this study is done during the pandemics of COVID-19, which is one of the influential for the breakage of the health system and some of these studies are done before the update of JNC-8. The variation for higher-than-expected levels may be due to the pandemics in which the patients must stay for longer at home without any follow-up with their medication. Patients mostly stay away from health facilities for a long period therefore, the patient fails to initiate treatments and needs frequent follow-up and type of medication and dose adjustment. When the patient visits the health facility infrequently, they fail to access regimen arrangement, and the patients are also unable to access bits of advice on non-pharmacological therapy.

The raised blood pressure level of patients with hypertension after initiating treatment may be due to many reasons. Resistant hypertension is one of the commonest characteristics of patients with failure to achieve the treatment target of patients with hypertension [25]. It may also be due to the measurement variation of uncontrolled blood pressure across the studies. Some studies do not consider chronic diseases like diabetes mellitus and chronic kidney diseases and the age of the participants. Clinicians better explore consider the reason for uncontrolled blood pressure and maintain frequent follow-ups than those patients [14].

Being male is one of the substantial factors for uncontrolled blood pressure. This is consistent with the longitudinal study conducted in Ghana [11] and a cross-sectional survey done in the United State [24, 26, 27] The variation of uncontrolled BP is attributed due to hormonal differences across males and females [28, 29]. The other reason for having uncontrolled blood pressure in males may be the male gender is associated with a higher number of chronic conditions [30]. Another possible explanation might be that gender differences may depend on the male patients with hypertension who experience poor adherence and compliance towards clinician counseling on the behavioral factors. Therefore, clinical physicians might need to pay more attention to male patients with hypertension, as they seem to be more vulnerable to becoming multimorbid in our study population.

Medication adherence is one of the main predictors of controlling high blood pressure for patients with hypertension. This is consistent with the study conducted in Nigeria and Gondar, Ethiopia [10, 15]. Medication adherence is one of the primary goals for blood pressure control. Patients who miss their prescribed dose will fail to attain the primary goals of antihypertensive treatment. Patients with poor medication adherence may take traditional medicine which may precipitate elevation of blood pressure [31]. Health care providers should provide any means of improvement for poorly adhered patients based on the reason for each patient.

Education is a predictor of uncontrolled blood pressure levels in patients with hypertension taking their medication for three months or more. Education is inversely associated with blood pressure control. Patients with hypertension with an educational level of secondary education and above can control their blood pressure [11]. This may be due to patients with high educational levels being able to adhere to clinicians' counseling on behavioral practice, medication adherence, and being able to understand the messages from health care providers [8]. Health care better to providers provide a tailored message in consideration of the educational level of the patients for attaining the target blood pressure.

Social support is a factor that contributes to blood pressure control for patients with hypertension taking drugs for three months or more. Patients with hypertension with low social support are at higher risk of uncontrolled blood pressure than patients taking antihypertensive treatment [32]. Low social support is the most commonest disorder in the pandemic of COVID-19 with a heavy burden of stress, loneliness, and depression [33, 34]. Stress results in an instantaneous sympathetic stimulus with a vasomotor response, resulting in a raised blood pressure [35]. These factors have hormonal influences on the level of blood pressure among patients with hypertension since one of the primary causes of hypertension is hormonal factors which are triggered through different factors.

### Limitation of the study

Since this was a cross-sectional study, it had the same limitations as other cross-sectional studies. In this study the outcome and exposure variables are measured at the same time, it is relatively difficult to establish causal relationships from a cross-sectional study. Patients may be biased to social desirability bias for doctors since adherence status was measured utilizing a document review.

## Conclusion

In this study, uncontrolled blood pressure in patients with hypertension on treatment is higher during the COVID-19 pandemic. Being male, poor medication adherence, and low social support are factors that contribute to uncontrolled blood pressure. Whereas, educational status was inversely associated with the blood pressure control status of patients with hypertension.

### Implications of the study

This study suggests that patients with hypertension on medication are still struggling to achieve target blood pressure control with a high level of low social support in the pandemics of COVID-19, so health care providers better screen patients for social support status, provide a coping mechanism for patients with low social support and refer patients to psychiatric clinics for additional psychological support.

## Data Availability

The manuscript contains all of the evidence that supports the findings. For further information upon reasonable request, the corresponding author may provide more comprehensive details and a dataset.

## Abbreviations

BP: Blood Pressure
JNC-8: The Eighth Joint National Committee
COVID-19: Corona Virus Diseases 2019
CI: Confidence Interval
APR: Adjusted prevalence Ratio
BMI: Body Mass Index
